# Bounding Quantum Correlations: The Role of the Shannon Information in the Information Causality Principle

**DOI:** 10.3390/e26070562

**Published:** 2024-06-29

**Authors:** Natasha Oughton, Christopher G. Timpson

**Affiliations:** 1 Somerville College, University of Oxford, Oxford OX2 6HD, UK; natasha.oughton@cs.ox.ac.uk; 2 Faculty of Philosophy, Brasenose College, University of Oxford, Oxford OX1 4AJ, UK

**Keywords:** Information Causality, Tsirelson bound, uncertainty, quantum correlations, Shannon information

## Abstract

The Information Causality principle was proposed to re-derive the Tsirelson bound, an upper limit on the strength of quantum correlations, and has been suggested as a candidate law of nature. The principle states that the Shannon information about Alice’s distant database gained by Bob after receiving an *m* bit message cannot exceed *m* bits, even when Alice and Bob share non-local resources. As originally formulated, it can be shown that the principle is violated exactly when the strength of the shared correlations exceeds the Tsirelson bound. However, we demonstrate here that when an alternative measure of information, one of the Renyi measures, is chosen, the Information Causality principle no longer arrives at the correct value for the Tsirelson bound. We argue that neither the assumption of particular ‘intuitive’ properties of uncertainties measures, nor pragmatic choices about how to optimise costs associated with communication, are sufficient to motivate uniquely the choice of the Shannon measure from amongst the more general Renyi measures. We conclude that the dependence of the success of Information Causality on mere convention undermines its claimed significance as a foundational principle.

## 1. Introduction

The principle of Information Causality, suggested by Pawłowski et al. [1], has received a significant amount of attention in the literature as a candidate principle to single out quantum correlations, following the research programme initiated by Popescu and Rohrlich. The aim of the programme is to use a black box approach, abstracting away from the underlying physical processes and characterising the physics just in terms of input–output properties, to investigate what principles might be proposed to capture quantum correlations from amongst other non-local (to be understood as Bell-inequality violating) correlations. One of a number of proposed principles, Information Causality, states that

the information gain that Bob can reach about a previously unknown to him data set of Alice, by using all his local resources and *m* classical bits communicated by Alice is at most *m* bits.

[1] (p. 1101)

Pawłowski et al. show that, with access to no-signalling correlations beyond the Tsirelson bound, which they describe as occurring “in a world in which particular tasks are ‘too simple’ and there exists implausible accessibility of remote data” (p. 1101), the Information Causality principle is violated. The authors suggest that in light of this derivation, Information Causality

…can therefore be used as a principle to distinguish physical theories from non-physical ones and is a good candidate for one of the foundational assumptions that are at the very root of quantum theory.

[1] (p. 1101)

As stated, the principle seems obvious: we would be unsurprised to learn that if Alice sends Bob *m* bits of information, the maximum amount of information that Bob gains from this is *m* bits, and if m=0, the amount of information gained is 0. However, the local resources available to Bob also include the parts of the correlations shared by Alice and Bob, represented by a shared black box. The principle also implicitly places a constraint on how much Alice and Bob can exploit these existing correlations to allow Bob to guess any set of Alice’s data set, which might be larger than *m* bits. (In fact, ref. [2] (p. 180) suggests the Information Causality principle would be more appropriately referred to as “informational neutrality of correlations”). Note that this somewhat undermines the intuitiveness of the above statement of the principle. In what follows, we suggest that there are more pressing problems for the justification of the Information Causality principle, further undermining its foundational significance.

Pawłowski et al. themselves do not offer much to motivate the choice of the Information Causality principle beyond its apparent intuitiveness, besides stating of its negation in their later paper: “things like this should not happen” [3] (p. 429). Cuffaro [4], noting this, suggests a potential avenue for justification by an appeal analogous to what he refers to as Einstein’s principle of mutually independent existence. However, in what follows, we question the strength of the analogy between the necessity of mutually independent existence for physical theory on the one hand, and non-trivial communication complexity for communication theory, on the other hand. More concerningly still, we point to a further problem that undermines the status of the Information Causality principle significantly. The authors quantify Bob’s information gain in terms of the Shannon mutual information between Alice’s data (which Bob is given the task of guessing some set of) and the resources available to him: his half of the shared correlations and Alice’s message. But as we will point out, the Shannon measure is not the uniquely natural uncertainty measure: the class of Rényi measures receives equally good justification. We argue that although the informal, non-mathematical, statement of the principle seems natural and intuitive, the mathematical formulation used in the derivation of the quantum bound rests on the choice of the Shannon information as the correct measure of information sent. We demonstrate that using a Rényi measure of uncertainty results in the Information Causality principle being no longer successful in singling out quantum correlations.

The argument proceeds as follows. Section 2 introduces the black box approach to singling out quantum correlations. In Section 3, we review the Information Causality principle, and in Section 4, discuss the extent of its justification. In Section 5, we consider the introduction of the Shannon measure, as well as a more general class of uncertainty measures. In Section 6, we show the Information Causality principle no longer results in the correct value for the Tsirelson bound when an alternative Rényi measure is used, and discuss implications in Section 7. Section 8 concludes.

## 2. Singling Out Quantum Correlations

### PR Boxes and the CHSH Game

The black box approach to capturing quantum correlations proceeds by abstracting away from the underlying physical processes by characterising the physics just in terms of input–output probabilities. The framework is usually introduced in terms of two player non-local games, consisting of spatially separated players, or parties, Alice and Bob, who work to maximise the probability of success of achieving a particular task, and a referee, who runs the game.

**Figure 1:**
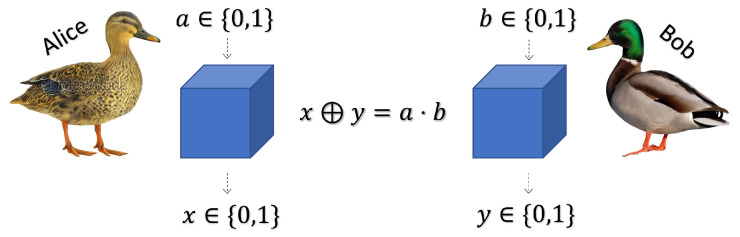
**Set-up of the CHSH game.** Alice and Bob, at a large distance from one another, input *a* and *b* and their respective boxes output *x* and *y*.

We consider the following set up. Two independent, uniformly chosen, random bits *a* and *b* are generated, a,b∈{0,1}, and *a* is sent to Alice whilst *b* is sent to Bob. Alice and Bob then both send a bit, x,y∈{0,1}, respectively, back to the source. Alice and Bob win the game by satisfying x⊕y=a·b, where ⊕ is modulo 2 addition (See Figure 1). This is satisfied if and only if the outputs are the same (00 or 11) if the inputs are 00, 01 or 10, and the outputs are different (01 or 10) if the inputs are 11. Alice and Bob play co-operatively, and aim to work together to get the highest probability of success. They are permitted to agree a strategy in advance, but once the game has begun, further communication is not allowed. Thus Alice does not know what bit Bob has received, and Bob does not know what bit Alice has received.

Making use of the CHSH [5] sum, K=〈00〉+〈01〉+〈10〉−〈11〉, it can be shown that Alice and Bob’s probability of success is given by $P_{\mathrm{CHSH}}=\frac{1}{2}\left(1+E\right)$, where $E=\frac{K}{4}$ is the normalised value of the CHSH sum.

From the CHSH result, we can now compare probabilities of success given access to the different resources available in different physical theories. Familiarly, the maximum probability of success for a classical strategy is given by $P_{\mathrm{CHSH}}^{C}\leq \frac{3}{4}$, corresponding to a value for $|K_{C}|\leq 2$, that is, $|E_{C}|\leq \frac{1}{2}$.

With access to the EPR state, Alice and Bob can exceed the classical limit for the probability of success, with a maximum value of $P_{\mathrm{CHSH}}^{Q}\leq \frac{2+\sqrt{2}}{4}\approx 85.4\%$. This corresponds to a value for $|K_{Q}|$ of $2\sqrt{2}$, and $|E_{Q}|=\frac{1}{\sqrt{2}}$. This value is known as the Tsirelson bound.

Note though, that the Tsirelson bound does not correspond to the maximum possible value of the CHSH sum: the CHSH inequality can hypothetically be violated further still if the four correlations in the CHSH sum are each at their maximum value of 1. In this case, the absolute value could be up to $P_{\mathrm{CHSH}}^{M}=1$.

Noting this, Popescu and Rohrlich speculate whether the constraint on quantum correlations might follow from no-signalling conditions (Although Popescu and Rohrlich describe these as imposing ‘relativistic causality’, we should take care to avoid conflating the above requirements with imposing relativistic constraints). Using the black box framework though, the answer is trivial. Popescu and Rohrlich, consider a particular black box, now known as a Popescu–Rohrlich, or PR, box, described by the probability distribution:

(1) $$P\left(x,y|a,b\right)=\left\{\begin{array}{cc} \frac{1}{2} & \mathrm{if}\;x⊕y=a\cdot b\\ 0 & \mathrm{otherwise} \end{array}\right..$$

The PR box is thus defined to satisfy the CHSH set of correlations described above,

(2) x⊕y=a·b,

as well as satisfying the no-signalling constraint: in fact, thinking in terms of a convex sets representation of probabilistic states, the correlations corresponding to PR boxes lie at the vertices of the non-signalling polytope. So quantum theory is not the unique theory that allows violation of Bell inequalities whilst still being compatible with no-signalling conditions: there also are ‘superquantum’ theories according to which correlations exist beyond the Tsirelson bound such that the no-signalling conditions hold but which allow violation of Bell-type inequalities. Thus, beyond asking why the correlations that we observe in the world are quantum rather than classical, a further question arises: why are they quantum rather than superquantum? What is the significance of the Tsirelson bound?

The question of whether imposing no-signalling and non-locality limits the correlations possible to quantum correlations, although answered negatively, has inspired a research project aiming to find a principle that successfully limits the possible correlations to those within the Tsirelson bound. Besides Information Causality, further proposed principles include Non-Trivial Communication Complexity (NTCC) [6], Non-Trivial Probabilistic Communication Complexity (NTPCC) [7] and No-Advantage for Non-Local Computation (NANLC) [8]. Two further principles take a different approach: Macroscopic Locality (ML) [9] and Local Orthogonality (LO) [10]. However, we may note that this research programme faces an overall challenge: Navascués et al. [11] define a set of correlations they call the ‘Almost Quantum’ set, and show that it strictly contains the set of quantum correlations, but is compatible with all of the information-theoretic principles above, with the exception of Information Causality. They also present evidence that strongly suggests the set is also compatible with Information Causality. We do not address this problem here, but see, for example [4] for discussion.

## 3. Information Causality and the Tsirelson Bound

### 3.1. The Information Causality Game

**Figure 2:**
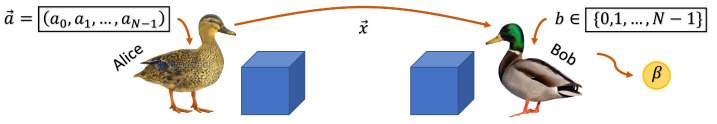
**The Information Causality game.** Alice receives a string of *N* bits, $\vec{a}$ and Bob a single variable b∈{0,⋯,N−1}. The task is for Bob’s guess, β, to be the $b^{th}$ bit of Alice’s string, after Alice sends message $\vec{x}$ of *m* bits to help.

In order to review the mathematically formal definition of the Information Causality principle, we begin by introducing the Information Causality game. We first consider the case where Bob must guess a single bit of Alice’s data set, after receiving a single classical bit from Alice, as depicted in Figure 2. The game can then be generalised to the case where Bob is tasked with guessing *m* bits of Alice’s *N* bits, with Alice sending *m* bits.

For the m=1 case, at each round, Alice has a string consisting of *N* random and independent bits, $\vec{a}=\left(a_{0},a_{1},\cdots ,a_{N-1}\right)$. Bob, at a distance from Alice, receives a randomly uniformly distributed variable b∈{0,1,2,⋯,N−1}. The task is for Bob’s answer, β, to be the $b^{\mathrm{th}}$ bit of Alice’s string. After the inputs have been provided, Alice is permitted to help him with this task by sending a single classical bit. Alice and Bob win a round if Bob correctly guesses the $b^{\mathrm{th}}$ bit for the round, and win the entire game if Bob guesses correctly each time over a succession of rounds.

Once the game starts, Alice and Bob cannot communicate, but may agree strategies and share resources in advance. It is assumed that the shared resources between Alice and Bob are no-signalling, since otherwise other communication channels could be opened. These shared resources can be described by black boxes, and may be correlated lists of bits, as would be the case classically, or a shared quantum state, or could be correlations stronger than possible according to classical or quantum theory. If no correlations existed between Alice and Bob, Bob can answer correctly if Alice happens to send the $b^{th}$ bit of her string, but when Alice sends some other $a_{k\neq b}$, Bob’s guess is at random, assuming there are no shared correlations.

If Alice and Bob have access to shared PR boxes, defined to saturate the CHSH inequality, Pawłowski and colleagues show that a strategy can be found that allows Alice and Bob to win the game in the m=1 case. This strategy can then be generalised to the m>1 case, where the task is for Bob to guess a set of *m* bits, after Alice sends an *m*-bit message. However, Alice and Bob cannot win the game in general with classical or quantum resources: it is shown that the point at which the Shannon information gained as a function of the probability of success of winning the game exceeds *m* bits is exactly the Tsirelson bound on quantum correlations. In what follows, we review this result.

There are two steps to the derivation given by [1]. Firstly, it is shown that, in the case where Alice receives two bits, that is, N=2, using a particular strategy, with access to PR boxes, it is possible to win the game, with certainty, each time. We can then consider what would happen if we replaced these PR boxes with black boxes characterised by their probability of simulating the PR box correlations, and therefore lead to Alice and Bob winning the defined game with a particular probability. This strategy can then be extended to the general case, where $N=2^{n}$, requiring *n* stages and *m* pyramids of boxes. Secondly, the point is calculated at which the probability of success of winning the game is high enough that the Information Causality condition is violated, which is shown to correspond to the Tsirelson bound.

Recall that Alice and Bob’s probability of success in reproducing PR correlations, maximising the CHSH sum, is given by

(3) $$P_{\mathrm{CHSH}}=\frac{1}{2}\left(1+\frac{K}{4}\right)=\frac{1}{2}\left(1+E\right),$$

where *K* is the CHSH sum and *E* is its normalised value, $\frac{K}{4}$.

With access to PR boxes, which correspond to E=1, Alice and Bob can win the game using their strategy. Replacing these PR boxes with boxes with values of |E|<1 allows us to consider whether classical correlations ($\left|E\right|\leq \frac{1}{2}$), quantum correlations ($\left|E\right|\leq \frac{1}{\sqrt{2}}$) or weaker than maximum superquantum correlations ($\frac{1}{\sqrt{2}}<E<1$) allow Alice and Bob to succeed at the Information Causality game over a number of rounds.

From this, we can calculate the probability that Bob’s final guess is correct given that Alice’s string is *N* bits long and that she is allowed to transmit an *m* bit message. The strategy requires *m* ‘pyramids’ of boxes, and *n* stages, where $N=2^{n}$.

It can be shown that, by following the strategy described by Pawłowski et al. [1], the probability that Bob’s guess is correct, and Alice and Bob win the game, is given by

(4) $$P_{W}=\frac{1}{2}\left(1+E^{n}\right).$$

This gives us the probability of success in winning the Information Causality game as a function of *E*. Note that just considering the probability of success does not yield the desired result: by this measure, entangled states can be shown to perform better than would be possible in the classical world. In order to single out quantum correlations, a ‘figure of merit’ is needed for which quantum and classical strategies achieve the same result, but which yields a different result for strategies involving correlations stronger than quantum. Thus, although the probability of success as a figure of merit has a simpler operational explanation, the Information Causality game considers as the figure of merit

(5) $$I\equiv \sum_{k=0}^{N-1}I\left(a_{k}:\beta |b=k\right),$$

where I(x:y) is the Shannon mutual information between random variables x,y. The Information Causality principle is then stated as

(6) I≤m.

### 3.2. Deriving the Tsirelson Bound

In their 2009 paper, Pawłowski et al. [1] proceed via a constraint on the mutual information between Alice and Bob’s datasets, associated with the probability of success figure defined in the previous section in order to calculate the value of *E* for which the Information Causality condition is violated. However, in the later discussion, we follow Bub’s (2012) derivation, which relates Information Causality directly to the binary information quantity. Al-Safi and Short [12] offer an alternative perspective on the Information Causality result: they show that the condition holds in any theory in which a so-called ‘good’ uncertainty measure can be defined, and point out that theories in which this condition holds are ‘very special’ within the class of generalised probability theories, and question whether quantum might in fact be unique in this respect. In contrast to the previous two, Al-Safi and Short’s derivation does not proceed via the probability of success.

Pawłowski et al. begin by deriving a theory-independent quantity that behaves like the mutual information measure for the classical and quantum cases. For a system made up of parts U,V and *W*, a symmetric and non-negative mutual information I(U:V) is defined that satisfies the following properties:

1. Consistency: If the subsystems *U* and *V* are both classical, then I(U:V) should be the same as the Shannon mutual information.
2. Data-processing inequality: Acting locally on one of the parts by any state transformation allowed in the theory cannot increase the mutual information. Thus if $V\to V^{\prime }$ is a permissible mapping between the systems, then it follows that $I\left(U:V\right)\geq I\left(U:V^{\prime }\right)$.
3. Chain rule: A conditional mutual information I(U:V|W) exists such that the following holds for all states and triples of parts: I(U:V,W)=I(U:W)+I(U:W|V).

These constraints are satisfied by the von Neumann measure, and the Shannon measure. Pawłowski et al. then show that for a mutual information as defined above, I≤m is a necessary condition for Information Causality.

In order to derive the lower bound on *I*, we begin with the definition of mutual information, and make use of the probability $P_{W}$, that Bob guesses correctly. We have that:

(7) $$I\equiv \sum_{k=0}^{N-1}I\left(a_{k}:\beta |b=k\right)=\sum_{k=0}^{N-1}\left(H\left(a_{k}|b=k\right)+H\left(\beta |b=k\right)-H\left(a_{k},\beta |b=k\right)\right).$$

Using the chain rule, and noting that Alice’s bits are uniformly random, so $h(a_{k})=1$, where $h\left(x\right)=-x\log_{2}x-\left(1-x\right)\log_{2}\left(1-x\right)$ is the binary information measure of *x*, we have:

(8) $$I\left(a_{k}:\beta |b=k\right)=1-H\left(a_{k}|\beta ,b=k\right).$$

Then, since the probability that $a_{k}=0$ is the same as the probability that $a_{k}⊕\beta =0$, and similarly for $a_{k}=1$, (that is, the probability that $\beta =a_{k}$), we have that $H\left(a_{k}|\beta ,b=k\right)=H\left(a_{k}⊕\beta |\beta ,b=k\right)$. Using the property of the Shannon measure that conditioning decreases information, it follows that $H\left(a_{k}⊕\beta |\beta ,b=k\right)\leq H\left(a_{k}⊕\beta |b=k\right)=h\left(P_{W}\right)$. Pawłowski et al. therefore show that

(9) $$I\geq N-\sum_{k=0}^{N-1}h\left(P_{W}\right).$$

Since $P_{W}$ is independent of *k*, this can be expressed as:

(10) $$I\geq N\left(1-h(P_{W})\right).$$

Then, since Information Causality requires I≤m, the resulting bound is:

(11) $$m\geq N\left(1-h(P_{W})\right).$$

Recall that $P_{W}=\frac{1}{2}\left(1+E^{n}\right)$. We therefore have a condition for Information Causality that depends on the likelihood of replicating PR boxes.

If Alice sends a single bit of information, i.e., m=1, Information Causality is violated if I>1, that is, if

(12) $$h\left(P_{W}\right)<1-\frac{1}{N},$$

or, since $N=2^{n}$, if

(13) $$h\left(P_{W}\right)<1-\frac{1}{2^{n}}.$$

Rather than proceeding via a constraint on the mutual information, as Pawłowski et al. do, Bub relates the constraint directly to a constraint on the binary information measure. In his derivation, Bub proceeds straightforwardly with the standard definition of the binary information.

Bub’s derivation considers the case where m=1, and proceeds as follows. If Alice sends one classical bit of information to Bob, then Information Causality stipulates that I≤m; that is, Bob’s information about Alice’s *N* bits can increase by at most one bit. Thus if Alice’s bits are uniformly random, Bob’s information about an arbitrary bit b=k in the list cannot increase by more than $\frac{1}{N}$ bits. That is, the binary Shannon information measure $h\left(P_{W}\right)=-P_{W}\log P_{W}-\left(1-P_{W}\right)\log\left(1-P_{W}\right)$ associated with Bob’s guess about an arbitrary bit in Alice’s list is at most $\frac{1}{N}$ closer to 0 (when $P_{W}=1$) from the value it would be at chance, 1 (when $P_{W}=\frac{1}{2}$). Thus we arrive at the following condition for Information Causality:

(14) $$h\left(P_{W}\right)\geq 1-\frac{1}{N}.$$

Substituting in Equation (4), we arrive at the result that whilst classical and quantum theories do not violate the Shannon measure version of the Information Causality principle, theories beyond the Tsirelson bound do (For further details, see [1] (p. 1103)). Pawłowski et al. take this to be an information-theoretic proof of the Tsirelson bound, which does not rely on the Hilbert space formalism.

## 4. Justifying the Information Causality Principle

Pawłowski et al. [1] claim that in virtue of its apparent success, Information Causality

…can therefore be used as a principle to distinguish physical theories from non-physical ones and is a good candidate for one of the foundational assumptions that are at the very root of quantum theory.

[1] (p. 1101)

However, for a principle to be held in this regard, we might reasonably expect it to be accompanied by a justification of the principle itself. Pawłowski et al. motivate its choice as follows

We show that in a world in which certain tasks are ‘too simple’ and there exists implausible accessibility of remote data, Information Causality is violated.

[1] (p. 1101)

Adding in a later discussion that “Things like this should not happen” [3] (p. 429).

We suggest that there are two major problems compromising claims for the justification of the choice of principle of Information Causality, however. First, we will discuss an attempt made by Cuffaro [4], who agrees that mere appeals to plausibility are unconvincing, and so instead attempts to provide an alternative justification for the Information Causality principle through Einstein’s principle of mutually independent existence. We will argue, though, that this approach has limited success. Secondly, we will argue that although the principle initially sounds intuitive, each strategy for deriving the Tsirelson bound rest on assumptions about the choice of information quantities involved that we argue are not sufficiently well justified.

Cuffaro [4] suggests an alternative justification for the principle of Information Causality, by appealing to the notion of mutually independent existence, a principle that Einstein regarded as necessary for the possibility of physical thought. Einstein argues for this as follows:

It appears to be essential for this arrangement of the things introduced in physics that, at a specific time, these things claim an existence independent of one another, insofar as these things ‘lie in different parts of space’. Without such an assumption of the mutually independent existence (the ‘being-thus’) of spatially distant things, an assumption which originates in everyday thought, physical thought in the sense familiar to us would not be possible. Nor does one see how physical laws could be formulated and tested without such a clean separation.

([13], translated in [14])

Inspired by the above, Cuffaro’s argument draws on posthumously published work by Demopoulos [15], who argues that what Cuffaro refers to as Einstein’s principle of mutually independent existence is (notwithstanding Bell inequality violation) satisfied in quantum mechanics, where it finds expression in the no-signalling principle. Cuffaro presents Demopolous’s argument that for an irreducibly statistical theory such as quantum mechanics, no signalling plays the role of local realism; it is a ‘surface level’ constraint on local facts associated with a system that requires that these facts be independent of the facts associated with other spatially distant systems. Cuffaro elaborates as follows: “no-signalling allows us to coherently treat systems in different regions of physical space as if they had mutually independent existences i.e., as quasi-closed systems in the sense described above” [4] (p. 273).

Cuffaro then argues that just as mutually independent existence is necessary for a system to be thought of as being subjected to empirical testing, so it is necessary within a communication theory to assume that an ‘operational distinction’ can be drawn between each of the parties involved. From this, he concludes that the concept of communication itself requires mutually independent existence, since otherwise “it is not at all obvious how one should begin to quantify the amount of information that is required to be sent from Alice to Bob in the context of a particular protocol” (p. 274). No-signalling assures this because it states that the marginal probability associated with a measurement on system $S_{L}$ at location *L* is independent of whether a measurement is performed on system $S_{R}$ at *R*.

Cuffaro then argues that Information Causality is furthermore necessary for mutually independent existence, stating that:

When Alice and Bob share maximally super-quantum systems [i.e., E=1], then after receiving [*m*] there is a sense in which Alice’s system can be said to be ‘a part’ of Bob’s system in the context of the game being played. For after receiving [*m*] Bob has immediate access to the value of any single bit of Alice’s that he would like. Alice’s bits may as well be his own for the purposes of the game.

[4] (p. 274)

The argument seems to be that using PR boxes, communication complexity is trivial. Cuffaro argues that although trivial communication complexity for distributed computation does not arise for all correlations beyond the Tsirelson bound, mutually independent existence is still violated, because the correlations shared by Alice and Bob before the game starts allow Bob to gain more information once the message has been sent than the quantity of information that Bob receives.

Cuffaro further argues that Information Causality is required for the practical theories associated with ‘resource’ or ‘control’ theories such as those concerning information, computation and communication (C.f. the discussion of thermodynamics as a control theory in, for example [16]). The argument is that it is necessary in a communication theory to be able to quantify the information transmitted from one entity to another, and this requires that it be possible conceptually to distinguish between the systems that are associated with each one of these entities on an operational level. Without this distinction, Cuffaro claims, it would not be possible to distinguish between distributed and localised computation, and the former is the subject of communication theory.

Cuffaro maintains that his argument is analogous to Einstein’s argument: Einstein takes it that the violation of the principle of mutually independent existence would make empirical testing of physical theories impossible; without it we could not empirically test whether some result concerns one region of space rather than another. But how strong is the analogy in truth, and can it bear the weight of justifying the Information Causality principle?

Consider: Physics is intended to be a universal and fundamental science; is the same true for communication theory, or communication theories? Would it matter if some physical things could not usefully be described in communication-theoretic terms? Suppose Cuffaro were right and that Information Causality’s holding were a necessary condition for describing things communication theoretically (though we are ourselves not entirely persuaded on this point). Unless there is some substantive reason for thinking that everything at all times must have a useful communication-theoretic description, or that no other than a communication-theoretic description of things were ever available, then we would have no reason to think that nature as a whole had to adhere to Information Causality.

Or again: The domain of quantum theory extends far further than just communication. Perhaps if quantum theory were solely a communication theory, then an argument along Cuffaro’s lines might be acceptable, but quantum theory is an autonomous theory that has wider applications than describing how communication can be carried out. Given this, there is no reason to require that the (allegedly) necessary conditions of communication theory’s applying be instantiated in nature. If we have available a physical description, communication-theoretic descriptions may fall where they will.

Where might similar reasoning might take us for other disciplines? Take economics for example. We would not be at all surprised to learn that some statements commonly accepted to be true about economics were not true universally. Of course, this is an extreme example, but it does not seem to be true that *all* science should apply universally.

Taking-up another concern, it is in a different way not clear that Cuffaro’s argument provides a justification for Information Causality. It seems only to rule out correlations that result in trivial communication complexity. As Cuffaro himself notes, it has not been proven that trivial communication complexity arises for all correlations that violate the Tsirelson bound, but only for values of *E* greater than $\frac{\sqrt{6}}{3}$, and not those above the Tsirelson bound $\frac{\sqrt{2}}{2}$ but below $\frac{\sqrt{6}}{3}$. Cuffaro considers whether “a little’ ambiguity may be tolerable for practical purposes” [4] (p. 276), concluding that there is more work to be done to close the gap. He also suggests that the status of Information Causality could be motivated if it were possible to link the “degree of violation of the principle and the degree of ‘superfluousness’ of the resulting theory of communication complexity” (p. 276), the idea being that weak violations of the Tsirelson bound would be more acceptable. The strength of Cuffaro’s proposal depends on the question one is seeking to answer. What is at issue here is why the physical theory that seems to correctly describe the universe we inhabit does not allow correlations beyond the Tsirelson bound. Cuffaro’s response would perhaps offer an explanation that would make such a theory more probable, but this does not seem a satisfactory response. However, for now, we will put such concerns to one side, before revisiting to show that this particular strategy will be (even) less successful than Cuffaro hopes.

## 5. Which Information?

Both derivations of the IC result described above proceed using the Shannon information, introduced by Shannon [17] in the context of communication.

For an information source associated with a set of possible letters or symbols $\left\{x_{1},x_{2},\cdots ,x_{n}\right\}$, each of which occur with probability $p(x_{i})$, the Shannon information quantity associated with the source is given by:

(15) $$H\left(X\right)=-\sum_{i=1}^{n}p\left(x_{i}\right)\log p\left(x_{i}\right).$$

Shannon’s noiseless coding theorem states that this corresponds to the optimum compression of an *N* symbol message, from Nlogn bits to NH(X) bits.

The Shannon quantity H(X) can also be introduced as a measure of uncertainty: the amount of spread of a probability distribution for a random probabilistic experiment, quantifying the uncertainty we have about the outcome of such an experiment.

The use of the Shannon information quantity to quantify the compression of information is taken to justify its role as a *bona fide* information quantity. However, as Shannon himself notes, the Shannon information is only one of a number of measures of uncertainty:

It is hardly to be expected that a single concept of information would satisfactorily account for the numerous possible applications of this general field.

[18] (p. 180)

A more general class of measures of uncertainty, U(P,μ) is discussed by Uffink [19], where *P* is a probability measure and μ a background measure. Uffink proposes a number of requirements that we expect uncertainty measures to fulfil:

- Invariance under permutations of the outcomes of probabilistic experiments
- Continuity
- Schur concavity

Heuristically, the requirement for Schur concavity ensures that our uncertainty measures track whether one distribution is more mixed or disordered than the other, and will agree that the uniform distribution is the most uncertain, and that the 0,1 distributions are the least uncertain.

The final requirement concerns the combination of probability distributions.

- The uncertainty associated with the combination of two probability distributions (P,μ) and (Q,v) over separate outcomes with coefficients α and β=1−α, for α≥0 is a function of U(P,μ),U(Q,v) and α.

Uffink then shows that the above requirements can only be satisfied by the following form of expression:

(16) $$U\left(P,\mu \right)=\chi^{-1}\left(\sum \mu_{i}\phi \left(\frac{p_{i}}{\mu_{i}}\right)\right),$$

where ϕ is a convex function and χ is a continuous decreasing function.

Choosing scaling conventions such that:

- When *P* is uniformly distributed over *A*, U(P,μ)=logμ(A) and
- U(P,cμ)=U(P,μ)+logc,

Uffink shows that the resulting class is of the form:

(17) $$H_{\alpha }\left(P,\mu \right)=\log U_{\alpha }\left(P,\mu \right),$$

Functions of the form $H_{\alpha }$ are called Rényi measures, and (when μ is the counting measure) can be expressed as

(18) $$H_{\alpha }\left(X\right)=\frac{1}{1-\alpha }\log\left(\sum_{x}p_{i}{\left(x_{i}\right)}^{\alpha }\right)$$

where *X* is a discrete random variable, with possible outcomes $x_{i}$ with probabilities $p(x_{i})$. In the limit α=1, the Rényi measure becomes the Shannon information.

Given that we have a more general class of uncertainty measures, we might ask why it is that we should privilege the Shannon information amongst them. Let us now consider some of the arguments given for the uniqueness of Shannon information, following [19,20].

Shannon [17] proposes a set of ‘reasonable’ requirements for a measure of uncertainty:

- *H* is continuous in the $p_{i}$.
- For equally probable events, *H* is a monotonic increasing function of n.
- If a choice is broken down into two successive choices, the original *H* is the weighted sum of the individual values of H.

In a later set of postulates due to Faddeev, this third requirement is made precise through the Faddeev grouping axiom. Uffink, though, argues that the grouping axiom is not naturally motivated, and should be rejected, because its adoption: introduces a conventional scaling definition into the axiomatic development; results in divergence problems when there is an unbounded number of items; and prevents extension to continuous distributions.

Shannon himself, though, did not attribute much importance to the derivation of his measure from the above requirements, and instead took its ‘real justification’ to be secured by its implications: its role in the noiseless coding theorem, and a number of ‘interesting properties’ that follow from the requirements. However, as noted by Uffink, the more significant of these properties arise as a result of imposing strict Schur concavity, and so Uffink’s more general class of uncertainty measures shares the same implications. Shannon’s argument thus fails to motivate the Shannon measure uniquely.

Uffink also considers whether a further constraint concerning joint experiments might single out Shannon information in conjunction with his own requirements. It turns out that the Shannon information is singled out if and only if

(19) $$H_{\alpha }\left(p\left(x_{i}\wedge y_{j}\right)\right)\leq H_{\alpha }\left(p\left(x_{i}\right)\right)+H_{\alpha }\left(p\left(y_{j}\right)\right).$$

It is usually argued that this inequality is a natural choice: when a joint distribution is replaced with the product of its marginals, then information is discarded and therefore uncertainty increases. However, as Uffink emphasised such reasoning is incorrect, since it is based on a false link between different senses of information. It does not follow from having lost information about correlations that the ability to predict the outcome of experiments is decreased (that our uncertainty necessarily increases). This is a tempting thought, but it equivocates between the notion of having information about a probability distribution, and the distinct notion of the degree of uncertainty one has about what the results of an experiment will be. It is the latter, not the former, which uncertainty measures quantify. Having lost information about the correlations, we have less information about the true joint probability distribution. But this does not mean our uncertainty need increase. Replacing a joint probability distribution with the product of its marginals (‘throwing away the correlations’) is not in general a doubly stochastic operation, so there is no requirement that uncertainty, as measured by a Schur concave function, will increase under this process; for some measures it may and for others it may not. It is only when two probability distributions are related by a doubly stochastic operation that all uncertainty measures will agree that one distribution has higher uncertainty than the other (See [19] Section 1.6.5 for further discussion and a concrete example where throwing away the correlations leads to a decrease of uncertainty, and [20] Appendix B for similar discussion).

Despite this, it is usually held that the Shannon information is the natural choice for an uncertainty measure because of the role it plays in the Shannon coding theorem. However, in work that has received comparatively little attention, Campbell [21] proposes a more general coding theorem in which the more general Rényi quantities $H_{\alpha }$ play an analogous role. Campbell notes that the derivation of the Shannon noiseless coding theorem involves the choice of code lengths so as to minimise the average code length, provided the code is uniquely decipherable, but there might be contexts in which we wish to minimise an alternative quantity. In contexts where the cost is an alternative function of the length, for example, if the cost of encoding and decoding equipment is significantly high, an alternative quantity might need to be minimised.

Campbell thus provides a characterisation of $H_{\alpha }$, the class of Rényi uncertainty measures, in the same way that the noiseless coding theorem provides a characterisation of $H_{1}$, the Shannon measure. This result is therefore a generalisation of Shannon’s noiseless coding theorem, and results in the Shannon version when the code length is the usual measure of mean length but as noted above, there are other ways in which the coding process can be optimised, each corresponding to a different Rényi quantity. From the perspective of the generalised coding theorem, then, the Rényi measures are equally as well justified as the Shannon information. Thus the choice of Shannon information from amongst the more general Rényi quantities depends on the choice of what one is seeking to optimise in a communication task.

The Shannon measure and related concepts, the Shannon conditional information and the Shannon mutual information, obey relations that make it very useful in manipulating concepts in information theory, such as non-negativity, monotonicity and the chain rule, amongst others. Only some of these are shared by the Rényi quantities in general.

The Rényi measures agree on extremal values, that is, if $H_{\alpha }=0$ holds for some value of α—for example, α=1, the Shannon measure—then it is also 0 for all values of α, and similarly for $H_{\alpha }=1$. In addition, Rényi quantities are positive and continuous; invariant under transformations of the outcomes of probabilistic experiments; and combine additively for independent probability distributions.

However, other features of the Shannon measure do not hold in general for Rényi measures. In particular, the strong subadditivity feature of the Shannon measure does not hold in general for Rényi measures (holding only for the α=1 quantity). In fact, Rényi measures in general are not subadditive at all except for the α=0 and α=1 quantities. The quantum Rényi measure (where we replace probability distributions with density operators) too does not satisfy subadditivity or strong subadditivity.

We also need to consider how one might define quantities analogous to the Shannon joint, conditional and mutual information for the Rényi measures. We can straightforwardly define a joint Rényi measure for two discrete random variables *X* and *Y*, with joint probability distribution $p_{XY}$ as follows:

(20) $$H_{\alpha }\left(XY\right)=\frac{1}{1-\alpha }\log\left(\sum_{x,y}p_{XY}{\left(x,y\right)}^{\alpha }\right).$$

However, for the case of analogous quantities for the Shannon conditional and mutual informations, the situation becomes more complex, since in both cases there are no commonly agreed-upon generalisations. A number of contenders have been proposed, as follows:

Cachin [22]:
  (21) $$H_{\alpha }^{C}\left(Y|X\right)=\sum_{x}p_{X}\left(x\right)H_{\alpha }\left(Y|X=x\right)$$
Jizba and Arimitsu [23]:
  (22) $$H_{\alpha }^{JA}\left(Y|X\right)=\frac{1}{1-\alpha }\log\sum_{x}\frac{p_{X}{\left(x\right)}^{\alpha }}{\sum_{x}p_{x}{\left(x\right)}^{\alpha }}2^{\left(1-\alpha \right)H_{\alpha }\left(Y|x=x\right)}$$
Renner and Wolf [24]:
  (23) $$H_{\alpha }^{RW}\left(Y|X\right)=\left\{\begin{array}{cc} \min_{x}H_{\alpha }\left(Y|X=x\right) & \mathrm{if}\;\alpha >1\\ \max_{x}H_{\alpha }\left(Y|X=x\right) & \mathrm{if}\;\alpha <1 \end{array}\right.$$
Arimoto [25]:
  (24) $$H_{\alpha }^{A}\left(Y|X\right)=\frac{1}{1-\alpha }\log\sum_{x}p_{X}\left(x\right)2^{\frac{1-\alpha }{\alpha }H_{\alpha }\left(Y|X=x\right)}$$
Hayashi [26]:
  (25) $$H_{\alpha }^{H}\left(Y|X\right)=\frac{1}{1-\alpha }\log\sum_{x}p_{X}\left(x\right)2^{\left(1-\alpha \right)H_{\alpha }\left(Y|X=x\right)}$$

These quantities originate in different contexts. The Arimoto quantity is proposed in the context of channel coding; the Cachin, Renner and Wolf, and Hayashi quantities are each proposed in the context of cryptography; and the Jizba and Arimitsu quantity is proposed in the context of multifractal analysis.

Ilić et al. [27] show that each of these definitions can be expressed as a single three-parameter quantity, given by:

(26) $$R_{\alpha }^{\beta ,\gamma }\left(X|Y\right)=g_{\gamma }^{-1}\left(\sum_{x}p_{X}^{\left(\beta \right)}\left(x\right)g_{\gamma }\left(H_{\alpha }\left(Y|X=x\right)\right)\right).$$

This general definition is positive, continuous, symmetric and permutation invariant for all α,β,γ. It is equal to the Rényi quantity for independent *X* and *Y*, equal to zero for X=Y and equal to the Shannon quantity for α=β=1. It also satisfies the following property: For three random variables, with the joint probability distribution $p_{X,Y,Z}$ and marginal distributions $p_{X},p_{Y},p_{Y,Z}$, we have:

(27) $$H_{\alpha }^{\beta ,\gamma }\left(Y,Z|X\right)\leq H_{\alpha }^{\beta ,\gamma }\left(Y|X\right).$$

The generalised measure therefore satisfies appropriate properties to be considered a conditional uncertainty measure. However, the following frequently used features of the Shannon conditional information do not hold in general, but only for particular choices of β and γ:

- The chain rule:
  (28) $$\begin{array}{cc} H_{\alpha }\left(X,Y\right) & =H_{\alpha }\left(X\right)+H_{\alpha }^{\beta ,\gamma }\left(Y|X\right) \end{array}$$
  (29) $$\begin{array}{cc} \; & \;=H_{\alpha }\left(Y\right)+H_{\alpha }^{\beta ,\gamma }\left(X|Y\right) \end{array}$$
- The weak chain rule:
  (30) $$H_{\alpha }^{\beta ,\gamma }\left(Y|X\right)\geq H_{\alpha }\left(X,Y\right)-\log m,$$
- Conditioning reduces the uncertainty (CRU):
  (31) $$H_{\alpha }^{\beta ,\gamma }\leq H_{\alpha }\left(X\right)$$
- The monotonicity property: if X,Y and *Z* form a Markov chain, then:
  (32) $$H_{\alpha }^{\beta ,\gamma }\left(X|Z\right)\leq H_{\alpha }^{\beta ,\gamma }\left(X|Y\right),$$

These definitions, along with their choices of β and γ, and the properties they satisfy, are summarised in Table 1.

Ilić et al. [27] also use their generalised conditional quantity to define a generalised mutual information quantity:

(33) $$I_{\alpha }^{\beta ,\gamma }=H_{\alpha }\left(X\right)-H_{\alpha }^{\beta ,\gamma }\left(X|Y\right).$$

This obeys the following properties fulfilled by the Shannon mutual information for all α,β,γ:

- $I_{\alpha }^{\beta ,\gamma }$(Y,X) is continuous with respect to $p_{X,Y}$
- If *X* and *Y* are independent, $I_{\alpha }^{\beta ,\gamma }$(X,Y) = 0
- If X=Y, we have $I_{\alpha }^{\beta ,\gamma }\left(X,Y\right)=H_{\alpha }\left(X\right)$

Again then, we can reasonably treat the general definition as an appropriate mutual information measure, but note that many of the typically used properties (including those utilised by Pawłowski et al.) do not, in general, hold.

We can therefore see that the choice of Rényi measure, and associated conditional and mutual information quantity, is dependent on the context in which it is applied, suggesting that the choice of Shannon information is a matter of convention rather than a natural choice.

## 6. Information Causality with Rényi Measures

Let us now review how the considerations above bear on the derivation of the Information Causality principle, and its claimed significance. Recall that Pawłowski et al.’s [1] derivation relies on assumptions about properties of information, which the Shannon and von Neumann information obey: subadditivity and strong subadditivity. However, we also saw earlier that these inequalities do not in general hold for the Rényi quantities, and therefore the derivation does not follow for Rényi quantities. As we saw in the previous section, the results of Uffink and Campbell imply that we have no prior reason to rule them out as appropriate uncertainty measures. We therefore conclude that the fact that Pawłowski et al.’s derivation cannot apply using Rényi uncertainty measures is a failing of the derivation, rather than a reason to reject the Rényi quantities as an appropriate measure of information. Thus, if Information Causality is to achieve a natural motivation, we need to find an alternative derivation that avoids reliance on these inequalities.

### 6.1. Bub’s Approach

We will begin by recalling that Bub’s heuristic derivation of the Information Causality condition gave the standard expression for when Information Causality would be violated:

(34) $$h\left(P_{W}\right)\leq 1-\frac{1}{2^{n}}.$$

Bub’s reasoning ought to apply equally if we were to choose a Renyi quantity instead of the Shannon quantity. In this case the condition would take the same form as above, simply with the Renyi entropy of the win-probability replacing the Shannon information:

(35) $$h_{\alpha }\left(P_{W}\right)\leq 1-\frac{1}{2^{n}}.$$

But as we will now show, if an alternative measure of uncertainty is chosen, the value of *E* at which Information Causality is violated changes.

A problem for the justification of the Information Causality principle arises if the point at which the inequality is violated changes using *any* uncertainty measure or other that receives the same support as the choice of Shannon information, for any value of *n*, and any value of α. Thus it suffices to show that a violation can be found for some value or other. For simplicity of calculation, let us take the Rényi uncertainty measure for α=2. The binary information measure for α=2 Rényi quantity is given by:

(36) $$h_{2}\left(P_{W}\right)=-\log\left(\frac{1}{2}\left(1+E^{2}\right)\right).$$

For $E=E_{\mathrm{PR}}=1$ (that is, with access to PR correlations), we obtain the expected result that the associated uncertainty is 0, so Bob knows with certainty what Alice’s bit is. For E=0, when no correlations exist between Alice and Bob, the uncertainty is 1, its maximum value, so Bob’s guess is at chance. Again, this matches our expectations given the nature of the game. However, for $E=E_{Q}=1/\sqrt{2}$, we obtain

(37) $$h_{2}\left(P_{W}\right)\approx 0.415\cdots <\frac{1}{2},$$

and the Information Causality principle is violated by *quantum* correlations, no longer correctly tracking the Tsirelson bound.

It thus follows that the value of the bound defined by Information Causality, as captured by Equation (35), depends on one’s choice of definition of uncertainty measure. This substantially undermines its significance as a candidate law of nature on two counts. Firstly, a principle that depends on a non-unique choice of measure is very difficult to justify. Secondly, the non-uniqueness of information measure means that it is hard to argue that the Information Causality principle points to some quantity out there in the world, and therefore the principle fails to identify a natural or objective quantity. Of course, the obvious reply to this comment would be to argue that the Shannon definition of information is the natural one to choose. However, as we previously argued there seems little motivation behind holding this position, and a supposedly fundamental principle should not be laden with mere convention, or pragmatic convenience.

One can also consider how the binary information measure, $h_{2}$, for the α=2 Rényi quantity (defined in Equation (36)) changes with other values of *E*, as presented in Table 2.

It can be seen from the above that if one were to consider, instead of the Shannon information quantity, the α=2 Rényi quantity, we would arrive at the result that Information Causality is violated at $E_{\alpha =2}\approx 0.644$. This substantially undermines Information Causality in its apparent plausibility and naturalness, since its derivation of the correct value for the Tsirelson bound turns out to depend on a somewhat arbitrary choice of uncertainty measure.

### 6.2. Pawłowski et al.’s Approach

So far we have been considering the case in which (35) is thought to express the conditions under which Information Causality would be violated, which will be so if Bub’s heuristic approach is applied to the general case. More rigorous, however, would be to seek to proceed via Pawłowski et al.’s approach in terms of mutual information (Equation (7) and following, above).

The difficulty here is, that as we have observed, there is no unique form for the mutual or conditional information once we move away from the α=1 case, and the properties of the information measures that Pawłowski et al. used to derive a simple expression for Information Causality violation in terms of the win-probability will no longer hold. This means that different measures will give different conditions for Information Causality violation, and moreover that there is no reason to think that any of them will provide a simple expression for the violation condition in terms of the win-probability. This means that it now becomes entirely unclear how strength of the correlations is to connect to satisfaction or violation of Information Causality. In other words we can no longer see how to connect the idea of Information Causality with a simple metric on correlations. Information Causality no longer seems operable to single out classes of strength of correlation. It might be felt that this point offers an argument in support of adopting the Shannon measure: it is only the choice of the Shannon measure which allows Information Causality to be turned into a functioning condition which can be applied to single-out certain classes of correlations. But this argument *assumes* that it is correct to begin with that Information Causality must have such a role. We, however, are casting doubt upon whether it really does, precisely because it is too sensitive to too many conventional and pragmatic considerations.

### 6.3. Al-Safi and Short’s Approach

A last hope for the Information Causality principle is an alternative derivation, due to Al-Safi and Short [12], which by-passes the inequality utilised in both Bub and Pawłowski et al.’s derivations. Al-Safi and Short show that the Information Causality result follows more simply from the existence of what they term a ‘good’ measure of uncertainty in a general theory (See also a similar observation due to Barnum et al. [28]). Note that Al-Safi and Short use the term ‘entropy’, but we opt to use the term ‘uncertainty measure’ here to avoid an association with the thermal notion.

This ‘good’ uncertainty measure is required to obey two conditions:

I Consistency: If system *X* is classical, H(X) reduces to the classical measure, $H\left(X\right)=H_{C}\left(X\right)$
II Evolution with an Ancilla: For any two systems *X* and *Y*, whenever a transformation is performed on *Y* alone,
  (38) ΔH(XY)≥ΔH(Y)

Condition I states that *H* describes the compression rate as the message length tends to infinity for classical data. Condition II can also be offered an intuitive understanding: it mandates that a local transformation can “generate more uncertainty than its effect on an individual subsystem would suggest, as it can destroy but not create correlations” [12] (p. 4). Alternatively, if a conditional uncertainty measure H(X|Y)=H(XY)−H(Y) is defined, this condition can be re-expressed as ΔH(X|Y)≥0.

Provided these conditions are satisfied, a mutual information can then be defined analogously to the quantum and classical quantities as:

(39) I(X:Y)=H(X)+H(Y)−H(XY).

The above guarantees that the chain rule and symmetry conditions are satisfied, and further, the consistency and data processing conditions are also satisfied since they follow from I and II, respectively. The existence of an uncertainty function that satisfies the Consistency and Evolution with an Ancilla conditions is thus sufficient to satisfy Information Causality. Reversing this statement, we have that in any theory in which Tsirelson’s bound can be violated, it is impossible to define an uncertainty in which the Consistency and Evolution with an Ancilla conditions are satisfied.

From conditions I and II, we can derive other standard properties usually associated with information quantities:

- Subadditivity:
  (40) H(XY)≤H(X)+H(Y)
- Strong subadditivity:
  (41) H(XYZ)+H(Y)≤H(XY)+H(YZ)
- Positivity of classical information: Uncertainty about the state of a classical system *X* can never be negative, even when it is conditionalised on an arbitrary system *Y*
  (42) SystemXisclassical⇒H(X|Y)≥0

Al-Safi and Short’s result allows the Information Causality result to be proven from the existence of an uncertainty measure that satisfies conditions I and II, then defining the mutual information and following the proof of [1]. Alternatively, they show that it can also be proven directly using the above listed properties of an uncertainty measure, which results in a more general version of the Information Causality principle.

However, note the similarity between the motivation behind condition II, Evolution with an Ancilla, and the argument discussed in Section 3 for the uniqueness of the Shannon information, which states that information is lost when a joint distribution is replaced with the product of its marginals. The same reply can be made here as earlier: as Uffink points out, when we lose information about correlations, it does not follow conceptually that our uncertainty increases about the result of an experiment.

More generally, we emphasise the need for caution when appealing to apparently intuitive motivations when considering information and uncertainty. Although Al-Safi and Short do not explicitly use the heuristic discussed above to argue for Condition II, there seems to be a suggestion that it is a natural condition to expect of an information measure given our pre-existing knowledge of classical and quantum physics. But there are other historical examples that demonstrate that such considerations can lead us astray. After all, if we had applied similar reasoning at a point where we only had knowledge of the classical Shannon information to consider what we would accept to be a good candidate for a quantum information, we might have ruled out the von Neumann information, since it behaves contra to how we might have expected had we previously only encountered classical information. For example, the conditional von Neumann information can be negative, whereas the conditional Shannon information can never be negative. We should be careful when extending our concepts to broader domains not to be overly guided by our intuitions when considering what constraints should carry over.

Al-Safi and Short utilise their definition of a ‘good’ measure to propose the following conceptual interpretation of the Information Causality principle: the principle states that the remaining uncertainty that Bob has about Alice’s bits after his guess is more than the original uncertainty about her inputs, minus the information gained by the message. They suggest that instead of considering Information Causality as a constraint on possible physical theories, instead it can be thought of as following from the existence of a ‘good’ measure of uncertainty in the theory. However, they point out that such ‘good’ measures, which share many of the properties of the classical information, are very rare within generalised probability theories, and therefore quantum physics is unusual in this regard. They raise the question of whether there are other theories for which such a measure can be defined, or whether this is unique to quantum theory, and suggest that the existence of such a measure may place stronger bounds on quantum theory than the principle of Information Causality alone.

## 7. Implications for Information Causality

The considerations above might lead one to question: if the choice of the Shannon information is a matter of convention or a matter of pragmatic calculational convenience, why does the derivation of the Tsirelson bound from the Information Causality principle depend on this choice? There are two possible routes for speculation: we might wonder whether the Tsirelson bound itself (that is, its specific value) is also somehow a matter of convention, or contains an element of convention, or it might be that some underlying property of the Information Causality principle corresponds to that used in Tsirelson’s original derivation. Here we highlight some potential avenues of inquiry.

First, we’ve already highlighted two alternative derivations, Al-Safi and Short’s and Barnum et al.’s, of the Information Causality principle through properties of an uncertainty measure in generalised probability theories. In addition to this, other authors have directly investigated the link between properties of uncertainty measures and the Tsirelson bound in the setting of generalised probability theories. For example, Dahlsten et al. [29] reveal that for any generalised probability theory that obeys the data processing inequality, the Tsirelson bound holds. Wakakuwa and Murao [30] also derive a similar result, by introducing a generalised mutual information (GMI): the optimal coding rate of a channel with classical inputs and general probabilistic outputs, which coincides with the quantum mutual information when the outputs are quantum. After noting that their generalised mutual information does not in general obey the chain rule, they go on to prove that Tsirelson’s bound follows after imposing the chain rule on the GMI. The authors conclude that the chain rule has some sort of operational significance, remarking the following:

This derivation shows that several laws of the Shannon theory, represented by the five properties of the mutual information, taken together impose a strong restriction on the underlying physical theory. If we take the GMI as the definition of the mutual information, it reduces to the statement that ‘a law of Shannon theory, namely the chain rule of the GMI, imposes a strong restriction on the underlying physical theory’.

[30] (pp. 16–17)

Note also that the chain rule plus the positivity of the mutual information (equivalent to the claim that conditioning reducing the uncertainty) together imply strong subadditivity. All of the derivations above make use of a set of linear inequalities known as Shannon-type inequalities, which hold for the Shannon information but, as noted, not for Rényi measures in general:
  - H(X|Y)=H(XY)−H(Y)≥0
  - I(X:Y)=H(X)+H(Y)−H(XY)≥0
  - I(X:Y|Z)=H(XZ)+H(YZ)−H(XYZ)−H(Z)≥0

Intriguingly, the same set of linear inequalities can also be used to derive information-theoretic Bell inequalities, analogous to CHSH inequalities, for information quantities [31,32]. Later work by Chaves [33], demonstrates that information-theoretic inequalities are necessary, and under certain circumstances, sufficient for standard Bell inequalities, and therefore it follows from the violation of information-theoretic Bell inequalities that standard Bell inequalities are also violated. This provides a further hint into what might underlie the link between the Information Causality principle, the Shannon information and the Tsirelson bound. Of course, none of this is conclusive, but it is suggestive of a connection in the mathematical formalism of particular uncertainty measures and that used to derive the Tsirelson bound.

## 8. Conclusions

We have seen that the Shannon statement of the Information Causality principle is apparently successful in re-deriving the Tsirelson bound, and is thus a candidate for a principle for singling out quantum correlations from amongst the no-signalling set.

Attempts to provide a justification for Information Causality in terms of the surprising conclusions following from the negation of an information-theoretic principle do not offer sufficient justification, since we might easily be led astray by following this route. Cuffaro’s attempt to provide a justification for Information Causality in particular by way of analogy with the principle of mutually independent existence was also shown to be unsuccessful, or at best considerably weaker than intended, given the lack of parallel between the impossibility of a communication-theoretic description and the impossibility of a physical description. However, a more pressing concern for Information Causality was also revealed: each of the three derivations of the Information Causality principle rest on insufficiently motivated assumptions about properties of information and uncertainty quantities.

We reviewed how the different concepts of information and uncertainty can be introduced, as well as the properties satisfied by various definitions. We argued that the appropriate quantity to use depends on the system and context in which one applies it. In particular, the Rényi measure satisfies all of the properties we might reasonably expect of a measure of uncertainty, as demonstrated by Uffink [19], and receives further justification through its role in a coding theorem, as shown by Campbell [21]. As a result, the choice to use the Shannon information from amongst this more general class of measures is largely a matter of convention and this significantly challenges the status of Information Causality to be a candidate foundational principle.

## Figures and Tables

**Table 1:** Summary of properties of proposals for conditional Rényi measures. The subscript α is omitted for brevity.

			Satisfies			
**Quantity**	** γ **	** β **	**Chain Rule**	**Weak Chain Rule**	**CRU**	**Monotonicity**
$H^{C}$	1	1	✗	✗	✗	✗
$H^{JA}$	α	α	✓	✓	✗	✗
$H^{RW}$	*∞*		✗	✓	✓(for α≥1)	✗
$H^{A}$	1	2−α	✗	✗	✓	✓
$H^{H}$	1	α	✗	✗	✓	✓

**Table 2:** Binary information measure for α=2 Rényi quantity, $h_{2}$, for other values of *E*.

*E*	$h_{2}$
0.6	0.556…
0.7	0.425…
0.644	0.499…

## Data Availability

No new data were created or analyzed in this study. Data sharing is not applicable to this article.
